# Polymyxin B haemoperfusion treatment for respiratory failure and hyperferritinaemia due to COVID‐19

**DOI:** 10.1002/rcr2.679

**Published:** 2020-11-01

**Authors:** Mayuko Ishiwari, Yuki Togashi, Hiroyuki Takoi, Ryota Kikuchi, Yuta Kono, Shinji Abe

**Affiliations:** ^1^ Department of Respiratory Medicine Tokyo Medical University Hospital Tokyo Japan

**Keywords:** COVID‐19, hyperferritinaemia, PMX‐DHP

## Abstract

A 69‐year‐old man with a history of type 2 diabetes and high blood pressure was diagnosed with coronavirus disease 2019 (COVID‐19). He had hyperferritinaemia and respiratory failure. Despite the initiation of favipiravir and high‐dose corticosteroid and ceftriaxone, his respiratory failure progressed and serum ferritin levels increased. After polymyxin B‐immobilized fibre column direct haemoperfusion (PMX‐DHP) therapy, there was improvement of the respiratory failure and hyperferritinaemia. We report the first case of COVID‐19‐induced hyperferritinaemia and severe respiratory failure successfully treated by PMX‐DHP.

## Graphical abstract

We report the first case of coronavirus disease 2019 (COVID‐19)‐induced hyperferritinaemia and severe respiratory failure successfully treated by polymyxin B‐immobilized fibre column direct haemoperfusion (PMX‐DHP) therapy.

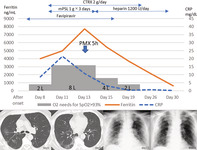

## Introduction

The severe acute respiratory syndrome coronavirus 2 (SARS‐CoV‐2) caused a worldwide pandemic that started in early 2020. An effective therapy has yet to be established for the acute respiratory distress syndrome (ARDS) associated with coronavirus disease 19 (COVID‐19). ARDS has a high mortality rate in hospitalized patients. Although the precise mechanisms of the severe respiratory failure caused by COVID‐19 remain unclear, hyperinflammation has been reported to play a prominent role in the pathogenesis of severe COVID‐19 pneumonia [1]. Hyperferritinaemia, which is categorized as hyperinflammation, has been reported to be an independent risk factor for the development of ARDS in COVID‐19 [2]. Ferritin can be a useful parameter to predict the severity and extent of inflammation. Moreover, ferritin may be actively secreted at infection sites and induce the expression of pro‐inflammatory and anti‐inflammatory cytokines, which might be pathogenic mediators [3].

We report a case of severe respiratory failure and hyperferritinaemia with COVID‐19 successfully treated with polymyxin B‐immobilized fibre column direct haemoperfusion (PMX‐DHP), which prevented the progression to ARDS and the need for invasive ventilation. Both oxygenation and inflammatory biomarker levels were dramatically improved by PMX‐DHP. PMX‐DHP may have a beneficial effect in respiratory failure with hyperferritinaemia caused by COVID‐19.

## Case Report

In April 2020, a 69‐year‐old man with a history of type 2 diabetes and high blood pressure presented with five days of dry cough and fever. A polymerase chain reaction (PCR) test for SARS‐CoV‐2 on nasal swabs was positive, and the patient was diagnosed with COVID‐19. On the eighth day after onset, he was admitted to the COVID‐19 ward of our hospital.

**Figure 1:**
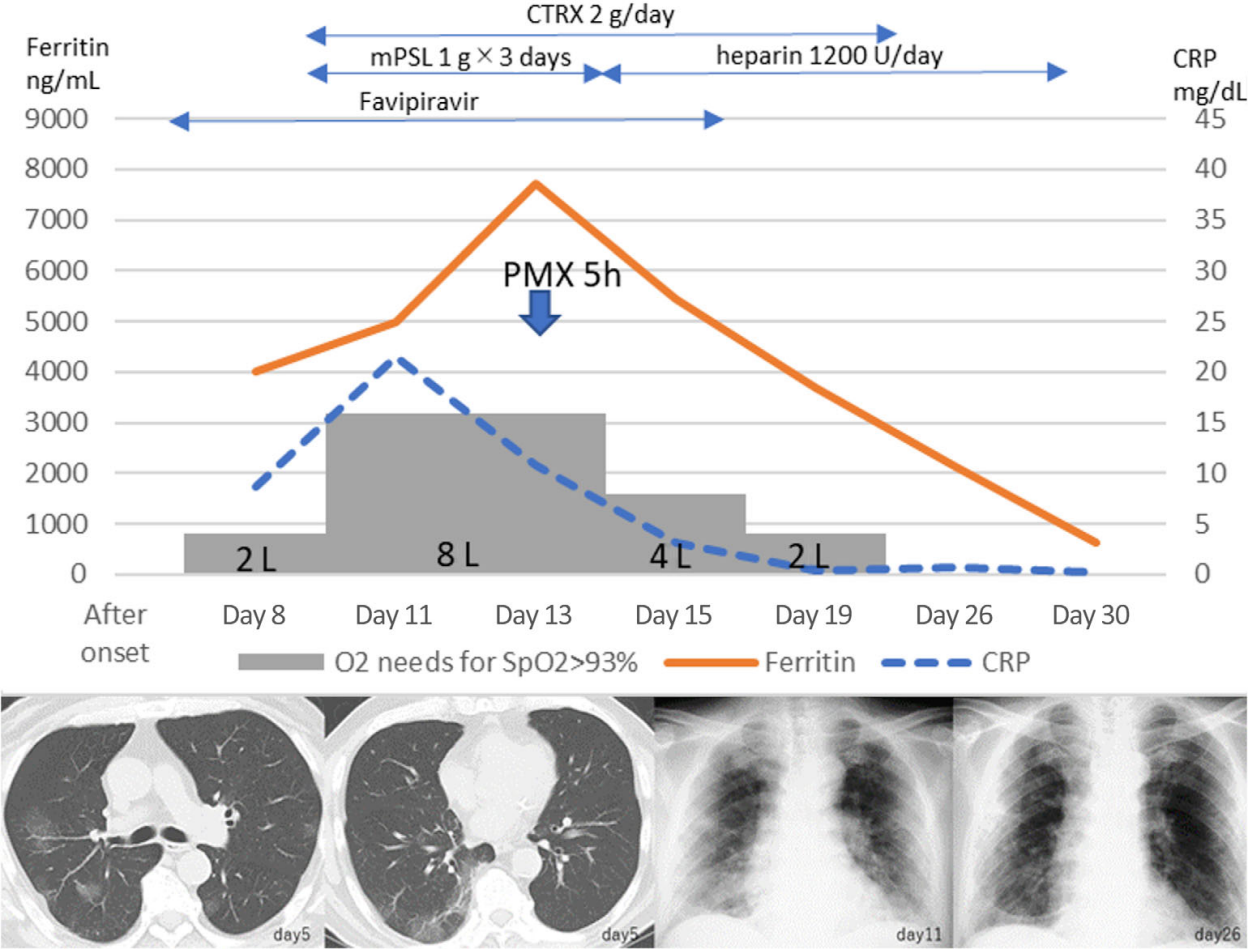
Timeline of disease course by day of hospital admission. After the administration of PMX‐DHP, the need for oxygen supplementation and the ferritin level decreased. CRP, C‐reactive protein; CTRX, ceftriaxone; day, after onset; mPSL, methylprednisolone; PMX‐DHP, polymyxin B‐immobilized fibre column direct haemoperfusion; SpO_2_, saturation of percutaneous oxygen.

On admission, he presented with a body temperature of 37.6°C, and 2 L/min oxygen via nasal cannula was started because his oxygen saturation (SpO_2_) was 89% on room air. Computed tomography (CT) scan of the chest revealed bilateral patchy ground‐glass opacity (GGO) consistent with COVID‐19 pneumonia (Fig. 1). Laboratory tests showed a white blood cell count (WBC) of 3700/μL (lymphocyte 1010/μL), and C‐reactive protein (CRP) was 8.7 mg/dL, ferritin was 4000.0 ng/mL, and d‐dimer was 1.17 μg/mL. Antiviral therapy with favipiravir was started on hospital day 1 (eighth day after symptom onset), with a first‐day dose of 1800 mg twice, and then 800 mg twice a day for two weeks.

On day 4 (11th day after symptom onset), sudden dyspnoea and oxygen saturation drop required an oxygen supplementation increase up to 8 L/min using a face mask. Laboratory tests showed an elevation of inflammatory markers: CRP was 21.5 mg/dL, ferritin was 7735.0 ng/mL, and d‐dimer was 1.81 μg/mL. X‐ray revealed bilateral deterioration with GGO (Fig. 1). High‐dose corticosteroid (methylprednisolone 1 g/day for three days) and ceftriaxone antibiotics were administered. Nevertheless, the patient's clinical condition, including oxygenation, worsened despite a slight decrease in CRP. On day 6 (13th day after symptom onset), 5 h of PMX‐DHP therapy was initiated to address the hyperinflammation. Post PMX‐DHP, the oxygenation and blood inflammation markers, including serum ferritin, were significantly improved. The progression to ARDS was halted and the need for intubation and mechanical ventilation was avoided. Serum ferritin levels promptly decreased to 2132 ng/mL after PMX‐DHP. Chest X‐ray showed that the bilateral infiltration gradually improved after PMX‐DHP (Fig. 1). No supplementary oxygen was required on day 19 (26th day of onset). The patient fully recovered from COVID‐19 symptoms and was discharged home after a 30‐day hospitalization.

## Discussion

COVID‐19 causes a spectrum of diseases ranging from mild symptoms (~80%) to respiratory failure (~20%), ARDS, and multiple organ failures. The leading causes of death are cytokine storm syndrome and ARDS, and 50% of patients with cytokine storm syndrome subsequently develop ARDS. Although the precise pathogenesis of severe COVID‐19‐induced ARDS remains unclear, hyperferritinaemia, which is categorized as hyperinflammation, is associated with severity and poor outcomes in COVID‐19 [1, 2]. Hyperinflammation is defined as follows: CRP concentration greater than 15 mg/dL; a doubling of CRP concentration within 24 h from a concentration of greater than 5 mg/dL; or a ferritin concentration of greater than 1500 ng/dL [1]. Ferritin levels at admission in COVID‐19 non‐survivors were reported to be around 1400 ng/mL [3]. In the present case, even when concomitant treatment with an antiviral agent and high‐dose corticosteroid were administered, serum ferritin levels were elevated (up to 7735 ng/dL) and no improvement in oxygenation was observed. We speculated that COVID‐19‐associated hyperinflammation or hypercytokinaemia induced severe respiratory failure, which could progress to ARDS. Next, we introduced DHP with PMX in order to remove inflammatory cytokines or mediators. Oxygenation and hyperferritinaemia were improved immediately after PMX‐DHP therapy. PMX was originally developed for the removal of endotoxins and is used to treat endotoxaemia. Recent reports have demonstrated the beneficial effect of PMX in severe influenza pneumonia, including both 2009pH1N1 and H5N1 [4, 5]. Hypercytokinaemia induced by 2009pH1N1 influenza was reported to be successfully treated with PMX, suggesting the potential efficacy of PMX to address hyperinflammation [4]. The removal of inflammatory mediators by PMX appears to be a promising treatment in patients at risk of developing ARDS due to COVID‐19.

In conclusion, we report the first case of COVID‐19‐induced hyperferritinaemia and severe respiratory failure successfully treated with PMX, which decreased the inflammatory markers and improved oxygenation. As a result, the patient's progression to ARDS was halted and the need for mechanical ventilation was avoided. Further studies are needed to determine the optimal timing of PMX in the disease course and its indication based on clinical parameters including hyperferritinaemia.

### Disclosure Statement

Appropriate written informed consent was obtained for publication of this case report and accompanying images.
